# Understanding of Stroke Risk Among Smokers in Saudi Arabia

**DOI:** 10.3390/medicina61061006

**Published:** 2025-05-28

**Authors:** Jaber S. Alqahtani, Abdulelah M. Aldhahir, Abdullah A. Alqarni, Saeed M. Alghamdi, Tope Oyelade, Fahad Nawar Alzaidi, Muath Abdulrahman Alamri, Ali Mohammad Kheder, Hussain Ibrahim Alsamdani, Ayadh Yahya AlAyadi, Rayan A. Siraj, Yousef S. Aldabayan, Ahmed M. Al Rajeh

**Affiliations:** 1 Department of Respiratory Care, Prince Sultan Military College of Health Sciences, Dhahran 34313, Saudi Arabia; 2 King Salman Center for Disability Research, Riyadh 11614, Saudi Arabia; 3 Respiratory Therapy Department, Faculty of Applied Medical Sciences, Jazan University, Jazan 45142, Saudi Arabia; 4 Department of Respiratory Therapy, Faculty of Medical Rehabilitation Sciences, King Abdulaziz University, Jeddah 22252, Saudi Arabia; 5 Respiratory Care Program, College of Applied Medical Sciences, Umm Al-Qura University, Makkah 24381, Saudi Arabia; 6 Division of Medicine, University College London, London NW3 2PF, UK; 7 School of Medicine, Keele University, Staffordshire ST5 5BG, UK; 8 Respiratory Therapy Department, King Faisal University, Al-Ahsa 31982, Saudi Arabia

**Keywords:** smoking, stroke, screening, risk factors, symptoms, awareness

## Abstract

*Background and Objectives*: Stroke is a common and fatal condition impacting millions worldwide. Understanding the risk factors, symptoms, and consequences at an earlier stage, specifically in highly at-risk populations such as smokers, is crucial for mitigating the stroke burden. *Materials and Methods*: A cross-sectional study was conducted in Saudi Arabia on more than 1000 smokers. The survey was randomly shared across the kingdom. Multiple logistic regression models were applied to assess the association and find the variables associated with poor stroke awareness. *Results*: The study included 1029 smokers, with 88% (904) being male. The majority (61%; 630) are between the ages of 18 and 30 years, with a university degree (84%, 862). Cigarettes (33.9%; 349), shisha (25.9%; 267), and e-cigarettes (40.1%; 413) were the most common smoking types. About 30% of smokers have never heard of stroke, and 44% were unaware that stopping smoking can prevent stroke. Smokers (55%) perceive smoking as the top risk factor, followed by heart disease (41%), and high blood pressure (40.8%), while 26% of the smokers did not identify any risk factors. Around 58% of the smokers did not identify any stroke symptoms. Only 20% of smokers were capable of identifying 50% of both stroke risk factors and symptoms. Around 8% reported all stroke-related consequences, whereas 44% were unable to identify any. Current smokers were more likely than ex-smokers to identify ≥1 stroke risk factor (OR = 1.95, *p* = 0.001), with no significant associations found for other variables. Males, university degrees, and current smokers were the only significant predictors for the identification of ≥1 stroke symptom. University education, current smokers, employment, and smoking types were significant predictors in identifying ≥1 stroke consequence. Compared to E-cigarette users, cigarette smokers exhibited twice the awareness of stroke consequences (OR = 2.01, *p* = 0.001), whereas shisha smokers demonstrated lower awareness (OR = 0.63, *p* = 0.01). *Conclusions*: Smokers’ awareness of stroke in Saudi Arabia is suboptimal. Targeted educational and smoking cessation initiatives are essential to address this gap and mitigate the impact of smoking-related strokes in Saudi Arabia.

## 1. Introduction

Globally in 2021, stroke was the third leading cause of death, resulting in 7.3 million fatalities, which constitutes 10.7% of all deaths [1]. In 2021, stroke was identified as the fourth leading cause of disability globally, with over 93 million prevalent cases [1]. Smoking constitutes a significant risk factor for strokes; however, it is a behavioral trait that can be modifiable to improve outcomes [1,2]. In Saudi Arabia, stroke is still one of the significant health issues, and it is considered the second most common cause of death in the country [3]. Stroke occurs at an annual rate of 29 per 100,000 people and is a leading cause of death and disability in the Kingdom [4]. However, there is still much work to be done in terms of raising awareness about stroke in the Saudi population, especially among smokers who are at higher risk [5]. Several studies have been conducted to determine the level of awareness of stroke risk factors and warning signs among the Saudi community and have shown varied results.

A cross-sectional study performed in the Qassim region demonstrated that 56.7% of hypertensive patients were smokers, which established this risk factor as the most dominant factor in this high-risk population [6]. This study showed an inadequate understanding of stroke risk factors and warning signs among those patients [6]. The necessity to focus on stroke awareness becomes evident because research indicates that 80% of strokes could be avoided through proper risk factor understanding [3]. The general population in Taif and Tabuk regions demonstrated insufficient knowledge according to two separate studies [7,8]. Another study in the Eastern region demonstrated that high school girls maintained a low understanding of stroke-related information, with 91.1% of participants scoring poorly [9]. Insufficient awareness of stroke risk factors and warning signs and the critical nature of timely intervention in stroke management may result in delays in seeking medical assistance [10]. Therefore, enhancing stroke awareness would facilitate the recognition of stroke symptoms and the prompt pursuit of medical care [11].

Indeed, the insufficient awareness of stroke risk factors, symptoms, and impacts among the public in Saudi Arabia is a significant contributor to the rising incidence and mortality rates [12]. Since smoking is one of the most important reversible risk factors for stroke, it is important to know the level of awareness of stroke risk among smokers in the Kingdom to design effective prevention programs [13]. Based on the Health Belief Model, smokers were specifically targeted in this study, as there are differences in the perception of risks between smokers and the general population in terms of susceptibility, severity, benefits and barriers [14]. Therefore, understanding the awareness of stroke risk among smokers will provide strong evidence for effort to improve quitting and reduce attributable burden.

Nevertheless, no single study has examined this issue within this specific vulnerable group of individuals in Saudi Arabia. This knowledge gap presents an opportunity for research to establish the current level of stroke awareness among smokers in Saudi Arabia. Such a study will be important in understanding the issue and help in designing specific education and awareness programs to reduce the incidence of stroke and, hence, enhance the health status of the people in the Kingdom.

## 2. Materials and Methods

### 2.1. Design and Participants

This research employed a cross-sectional survey design to gather data from a representative sample of the smoker population in Saudi Arabia. We used convenience sampling due to the convenience-based and exploratory nature of the study. Given that our study seeks to obtain preliminary insights rather than to rigorously test a hypothesis with strict generalizability, a formal power calculation was not conducted. This approach is commonly utilized in exploratory studies to inform subsequent rigorous studies. The study sample included male and female smokers, 18 years of age and older, with a minimum sample size of 500 participants.

### 2.2. Measurement Tool

We utilized a modified previously published questionnaire hosted at Google Forms to measure awareness of stroke risk among smokers [15,16]. The questionnaire was initially developed in English and then translated into Arabic and had two parts. The first part of the questionnaire gathered information on the socio-demographic characteristics of the respondents, such as gender, age, area of residence, marital status, highest education completed, occupation, monthly household income, and smoking status. The second part of the survey focused on stroke awareness, risk factors, early warning signs, and consequences of stroke. The research team has assessed the survey’s face and content validity. No pilot testing was needed, as the survey was widely used in Arabic speaking countries with published evidence, which validates its usefulness.

### 2.3. Setting and Ethical Consideration

We distributed the survey in Arabic, the native language of Saudi, using official social media platforms (between 20 December 2024 and 15 March 2025). Participants received information about the study’s purpose, estimated survey completion time, data confidentiality, and voluntary participation. No incentives were provided. Before starting the questionnaire, participants were asked to give their consent. Ethical approval was secured from the Institutional Review Board of Prince Sultan Military College of Health Sciences (IRB-2025-RC-006).

### 2.4. Data Analysis

Data were automatically gathered via the hosting platform and exported into an Excel file. One author verified the data to minimize errors, while another cross-confirmed them. We used descriptive analysis to analyze responses. Multiple logistic regressions were employed to evaluate the associations among variables, with the dependent variables being the identification of stroke risk factors (≥1), stroke symptoms (≥1), and stroke consequences (≥1). The responses were analyzed using SPSS version 25 (IBM Corp., Armonk, NY, USA). A *p*-value ≤ 0.05 was considered statistically significant.

## 3. Results

The study sample consists of 1029 smokers, with 88% (904) male. The majority belong to the 18–30 age group (61%; 630), and (84%, 862) hold a university degree. Regarding smoking habits, (67.8%; 698) are current smokers, with preferences divided among cigarettes (33.9%; 349), shisha (25.9%; 267), and e-cigarettes (40.1%; 413). Among e-cigarette users, nicotine concentrations vary, with 29.3% using 20–30 mg and 28.1% opting for 40–50 mg (Table 1). Consumption patterns reveal that 31.2% use e-cigarette liquid within 3–4 weeks, while 29.8% take longer than a month. High nicotine dependence on e-cigarettes was reported in 9% (36) of users.

### 3.1. Familiarity with Stroke

**Figure 1:**
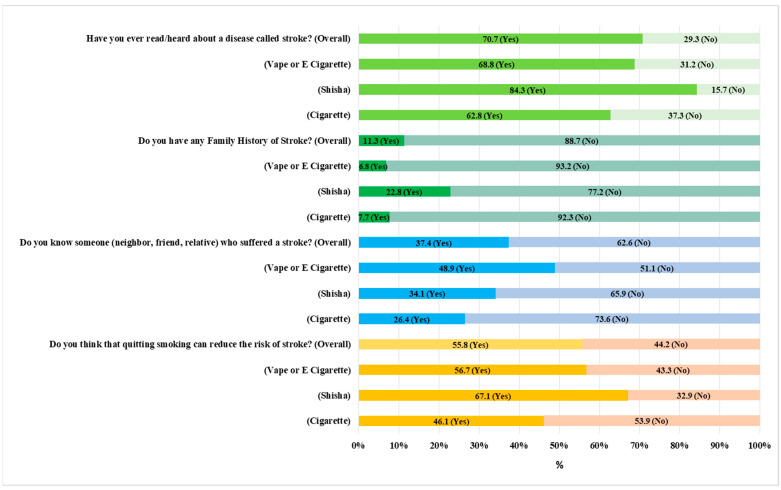
Familiarity with stroke by type of smoking (N = 1029).

We asked the participants four questions to understand their familiarity with stroke and Figure 1 shows their responses by different smoking types. The results indicate that about 30% of the smokers have never come across or read about stroke, while only 11% have a family history of the condition. Stratification by smoking types demonstrated that 37% of cigarette smokers were unaware of stroke, compared to 31% of e-cigarette users and 15.6% of shisha smokers. About 37% of the respondents know someone who has had a stroke, a neighbor, a friend, or a relative. Yet, 44% of the respondents were not aware that quitting smoking can help prevent stroke. Analysis by smoking types revealed that 54% of cigarette smokers were unaware that cessation can reduce the risk of stroke, followed by 43% of e-cigarette users.

### 3.2. Identification of Risk Factors of Stroke 

**Figure 2:**
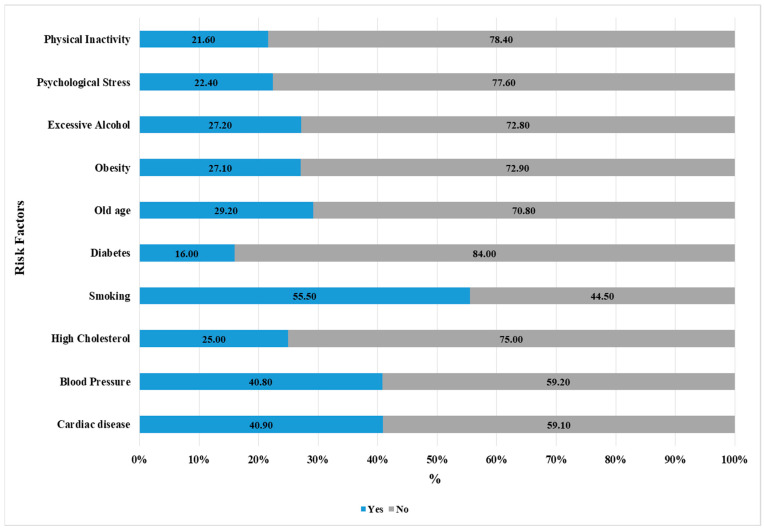
Identification of risk factors of stroke (N = 1029).

The results demonstrate how smokers perceive different stroke risk factors. It shows that smokers recognize smoking as the most identified risk factor (55%), while cardiac disease (41%) and high blood pressure (40.8%) follow closely, indicating fair cardiovascular health awareness. Smokers recognize lifestyle-related stroke risks including high cholesterol (25%) and obesity (27%) and excessive alcohol consumption (27%) but at lower rates, which indicates knowledge gaps about metabolic stroke causes. Old age (29%), diabetes (16.0%), and behavioral stroke risk factors including psychological stress (22.4%) and physical inactivity (21.6%) receive less recognition (Figure 2). Only 4.5% (46) of smokers have acknowledged all 10 risk factors, whilst 26% (270) were unable to identify any of them (Table 2).

### 3.3. Identification of Stroke Symptoms

**Figure 3:**
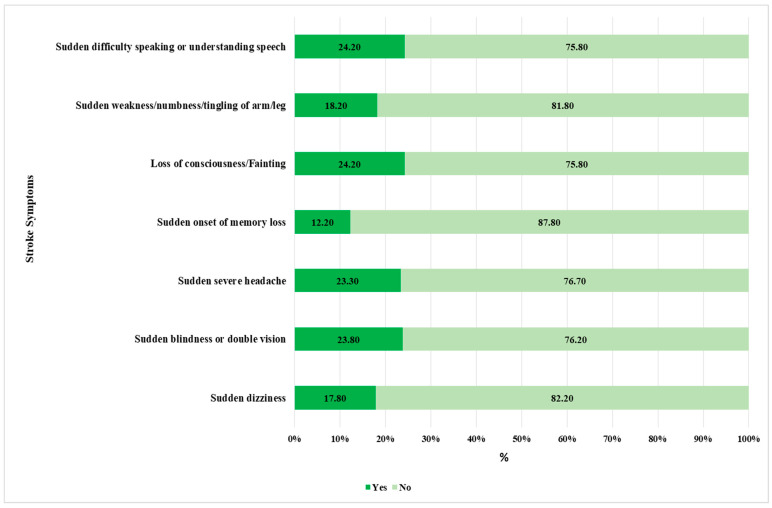
Identification of symptoms of stroke (N = 1029).

The most recognized symptoms include loss of consciousness or fainting (24.2%) and sudden difficulty speaking or understanding speech (24.2%) and sudden blindness or double vision (23.8%) and sudden severe headache (23.3%) (Figure 3). The awareness level is found to be low for sudden weakness, numbness, or tingling in the arm or leg (18.2%) and sudden dizziness (17.8%). The least recognized symptom is the sudden onset of memory loss (12.2%), which shows that there is a lack of understanding of how stroke impacts cognitive function. Only 13.7% (141) of smokers have acknowledged all symptoms, whereas 57.6% (593) have failed to identify any of them (Table 2). We noticed that only 20% of smokers were capable of identifying 50% of both stroke risk factors and symptoms.

### 3.4. Awareness of Stroke Consequences

**Figure 4:**
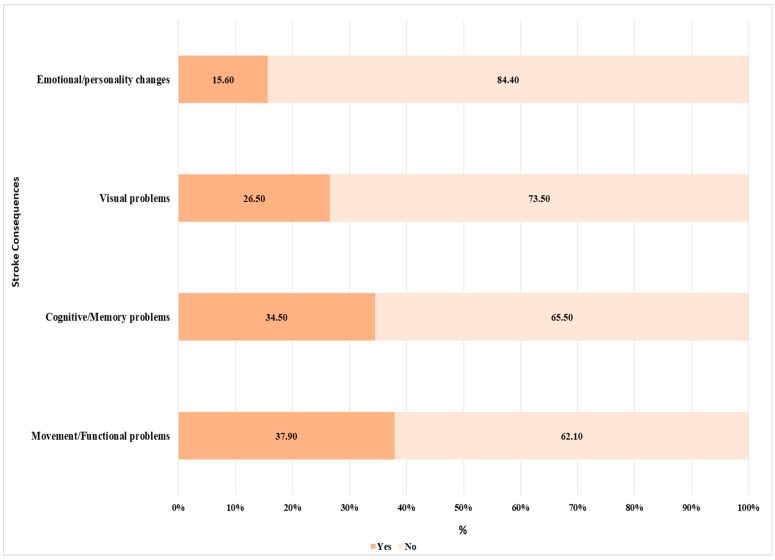
Awareness level of stroke consequences (N = 1029).

The findings reveal that Movement/Functional problems (37.9%) and Cognitive/Memory problems (34.5%) are the most recognized aftereffects of stroke (Figure 4). The awareness of visual impairments was 26.5%, higher than emotional and personality changes (15.6%), the least recognized effect. Around 8% reported all stroke-related sequelae, whereas 43.7% (450) were unable to identify any (Table 2).

### 3.5. Association of Risk Factors, Symptoms, and Consequences of Stroke with the Socio-Demographic Characteristics

The participants from the Central Region demonstrated more than 2-fold the ability (OR = 2.42, *p* = 0.001) to identify ≥1 stroke risk factors when compared to smokers from the Western Region. Current smokers were more likely to identify at least one risk factor (OR = 1.95, *p* = 0.001) than ex-smokers. There were no statistically significant relationships found for gender, residence area, educational level, job status, and type of smoking.

Regarding identifying stroke symptoms, the results showed that men had a 2.88 times higher chance of identifying stroke symptoms than women (*p* = 0.001). Those with a university education had 47% higher awareness than those with a school education only (OR = 0.53, *p* = 0.001). Current smokers were 2.68 times more aware than ex-smokers (*p* = 0.001), with no statistically significance differences in other variables.

Concerning identifying stroke consequences, central and eastern regions were more aware of the stroke consequences than the western, with OR of 3.16 and 1.52, respectively (Table 3). University-educated smokers had 36% greater awareness than those with school education (OR = 0.64, *p* = 0.02), and employed smokers were 1.40 times more aware than non-employed (*p* = 0.03). Further, current smokers had 2.36 times greater awareness of stroke consequences than ex-smokers (*p* = 0.001). Compared to E-cigarette users, cigarette smokers showed a higher awareness of stroke consequences (OR = 2.01, *p* = 0.001), while shisha smokers had lower stroke consequence awareness (OR = 0.63, *p* = 0.01).

## 4. Discussion

In this study, we present, for the first time, the awareness of stroke risk factors, symptoms, and sequelae among a random population of adult smokers from the Kingdom of Saudi Arabia. We also investigated the factors associated with this awareness among the study population. Participants were stratified based on the types of smoke used including cigarette, shisha, or e-cigarette to understand any links between these and awareness about stroke.

In terms of awareness, more than 2/3 of the smokers (70%) were familiar with stroke, with more than 1/3 (37%) knowing someone who developed a stroke and a few smokers (11%) having a family history of stroke. This level of smokers’ awareness is lower than those reported in previously published studies involving the general population. Specifically, in a study of the general, mainly urban-dwelling Saudi population, Alzayer et al. reported an awareness of stroke as a brain disease among participants where 90% have this knowledge [17]. Similarly, in a survey of Lebanese participants, Malaeb et al. reported a higher familiarity whereby 94% of participants explicitly responded as being aware of stroke. Further, the proportion of participants in this study who either know someone with a stroke or have a family history of stroke was also higher at around 69% and 28% respectively [15]. Also, a cross-sectional survey of the Jordanian general population corroborated the higher awareness in Lebanon, where Barakat et al. reported 95% awareness, with 31% and 77% of the participants reporting a family history of stroke and personally knowing someone with stroke, respectively. While these studies show higher awareness in the general population, our study focuses exclusively on smokers, which may explain the relatively lower awareness level.

Overall, more than half (55%) of all participants recognized smoking as a risk factor for stroke, with 41% and 40.8% recognizing cardiac disease and HBP as risk factors. Metabolic risk factors (including high cholesterol, obesity, and alcohol consumption) were less recognized as major risk factors in this study. While old age was recognized by around one-third of participants, diabetes and behavioral risk factors were less commonly identified by participants. Less than 5% of the participants were aware of all 10 risk factors listed in the questionnaire, highlighting an overall knowledge gap. While this is a similar level to that reported in a previous study among the general Saudi population [17], our result shows a relatively higher awareness of smoking as a risk factor for stroke among adult smokers in Saudi Arabia compared with previous studies from other countries within the region. For instance, a similar survey of the Jordanian and Lebanese populations reported a relatively lower proportion of participants were able to make the risk–disease link, with both studies reporting percentage of ~34% and 27%, respectively [15,16]. A significant proportion of participants in this study (44%) were not aware that quitting smoking can prevent stroke, with more than half of cigarette smokers (54%) not aware that quitting could reduce the risk of stroke. This is an interesting finding, given that more than half of the study participants recognized smoking as a risk factor for stroke. This dissonance in harm perception has been previously described and highlights the gap in literacy or awareness campaigns, showing that while smokers may believe that smoking is harmful, they do not perceive the potential benefits of quitting smoking [18,19]. Cognitive dissonance may explain this; smokers minimize the value of quitting to avoid the discomfort of knowing smoking is bad. Despite knowing the risks, optimism bias may make smokers think poor health outcomes are unlikely to happen to them, decreasing their desire to quit [14]. This provides a case for targeted, personalized, bottom-up approaches to improve health literacy, especially about the risk of stroke and other cardiovascular diseases attributable to smoking in Saudi Arabia.

The awareness of stroke symptoms was also relatively low among participants, with less than a quarter of participants being aware of the main symptoms of stroke, including fainting, loss of speech, problems with vision, and sudden severe headaches. Participants were even less familiar with other symptoms, including memory loss, numbness, tingling arms or legs, and dizziness, among others. Regarding the post-stroke sequelae, participants’ awareness was low, with around 44% of participants unable to identify any of the listed outcomes. The awareness herein is generally lower compared to the general population, as reported in previous studies [17,20]. For instance, Alzayer et al. reported that more than 50% of the general study population were aware of early symptoms of stroke, including sudden loss of consciousness (fainting), severe headache, and dizziness, with around 80% being aware of sudden loss of speech or understanding [17].

In terms of the likelihood of identifying risk factors for strokes, results show that living in the central region of Saudi Arabia and being a current smoker increases the odds of being able to identify one or more risk factors. These findings are contrary to a previous study that showed no association between the region of residence and awareness of risk in the Saudi general population. Also, this study does not show any association between awareness of risk factors and a history of smoking, as reported herein [17]. Similarly, the study on the general population in Lebanon showed no association between residence area or history of smoking and awareness of risk factors [15]. However, the study among Jordanians, which shows a significant association between residence area and risk factor awareness, with urban residents being more aware [16], supports our findings.

In terms of identifying the early symptoms of stroke, male gender, university degree, or being a current smoker were all significantly linked with increased awareness about the symptoms of stroke. Similarly, participants living in central or western regions, those with university education, those who are employed, or those who are current smokers were relatively significantly more likely to be aware of the sequelae of stroke. The importance of higher education as a protective factor against various diseases is well established in the literature [21,22,23]. This effect is generally modified by the higher level of health literacy and better information-seeking behaviors, attributed to a higher level of education [24]. As reported above, participants with higher education were more likely to identify early symptoms as well as understand the outcomes/sequelae of stroke. Contrary to previous studies from the region where no association was reported [15,16], our results show that being male and being a current smoker were linked with awareness about the early symptoms as well as the sequelae of stroke. This could be linked to differences in cultural information access and education level. The links between being able to identify the consequences of stroke and region of residence are supported by findings by Barakat et al. from Jordan [16], but work by Malaeb et al. on Lebanese participants did not show such a link [15]. A similar link with employment status was supported by Malaeb et al. [15] but not the study by Barakat et al. [16]. Finally, cigarette smokers were significantly more likely to be aware of stroke consequences compared with e-cigarette and shisha users. The higher awareness among males and cigarette smokers may be related to the Health Belief Model. Specifically, males are generally more susceptible to strokes [25,26]. Thus, a perception of higher susceptibility and severity among men, as well as the fact that most anti-smoking campaigns against cigarettes are targeted at males, may explain the higher awareness among this sub-population. This highlights the need to target this group for optimum outcomes in reducing smoking burden.

This study has various limitations. First, the cross-sectional design of this study means while associations can be made, it is not feasible to establish causality or reverse causality among the variables analyzed. Second, the sample size, while larger than previous studies in the region, is based on an online convenience sampling method and may not entirely represent the general population of smokers in Saudi Arabia, nor could the findings from the national population be generalized to other countries. Particularly, selection bias may result from the online sampling method, which may result in digital exclusion and skewing the data towards younger, educated participants with access to technology. However, targeting the high-risk population (i.e., current smokers) allows targeted inference that could be used to design targeted interventions to reduce the burden of smoking-related stroke both in Saudi and the entire Middle Eastern region. The surveys depend on recollections that may result in recall bias and may lead to data omission, affecting the reliability of the results as well as generalizability. Finally, the psychometric properties of the survey questionnaire may not capture all aspects of risk awareness and future study may need to reassess it within different regions. The survey questions are not exhaustive in asking about all the factors or determinants of awareness of stroke risk, symptoms, and sequelae among smokers, potentially leading to residual confounding bias. However, this study remains the largest study of its kind, targeted at smokers in the Kingdom of Saudi Arabia with participants randomly selected across various regions of the country based on a questionnaire that has been previously used within the Arabic-speaking regions.

## 5. Conclusions

Awareness of risk factors, early symptoms, and sequelae of stroke among smokers in the Kingdom of Saudi Arabia is relatively low. This awareness is generally affected by education level, region of residence, gender, employment status, and smoking behavior. Future studies should employ an even larger sample size across different countries to understand the awareness of stroke and the determinants of such awareness among current smokers. All in all, the result of this study provides usable findings for public health experts and organizations in designing targeted campaigns and crisis management strategies to reduce smoking. In particular, carefully designed, evidenced-based mass campaigns, smoking cessation programs based on Health Belief Models and integration of stroke education into primary care services should be implemented.

## Figures and Tables

**Table 1:** Characteristics of the participants (n = 1029).

Demographic Characters	N (%) or	
	Mean ± SD	
**Gender**		
Male	904	87.9
Female	125	12.1
**Age group**		
18–30 Year	630	61.2
31–40 Year	227	22.1
41–50 Year	90	8.7
>50 Year	82	8
**Living Area**		
Rural	52	5.1
Urban	977	94.9
**Marital Status**		
Married	274	26.6
Single	755	73.4
**Regions**		
Western	397	38.6
Eastern	348	33.8
Central	144	14
Southern	66	6.4
Northern	74	7.2
**Educational Status**		
School	167	16.2
University	862	83.8
**Employment Status**		
Employed	559	54.3
Unemployed	470	45.7
**Income Level**		
Less than 10,238	704	68.4
More than 10,238	325	31.6
**Smoking Status**		
Current Smoker	698	67.8
Ex-smoker	331	32.2
**Types of smoking**		
Cigarette	349	33.9
Shisha	267	25.9
Vape or E-cigarette	413	40.1
Pack Per Year (Cigarette)	13.26 ± 17.96	
Pack Per Year (Shisha)	24.92 ± 70.90	
**Nicotine used in E-cigarette liquid**		
0 mg	27	6.5
2 to 18 mg	63	15.3
20 to 30 mg	121	29.3
30 to 40 mg	86	20.8
40 to 50 mg	116	28.1
**Bottle of e-cigarette consumption (used ≥20 mg in a week)**		
In one week	65	15.7
2 to 3 weeks	96	23.2
3 to 4 weeks	129	31.2
Longer than one month	123	29.8
**High Nicotine dependence for E-cigarette**	36	9%

**Table 2:** Number of stroke risk factors, early symptoms, and consequences that were identified by the participants.

Variables	N (%)	
**Identification of risk factors for stroke**		
Zero	270	26.2
One risk Factor	55	5.3
Two risk Factors	189	18.4
Three risk Factors	95	9.2
Four risk Factors	139	13.5
Five risk Factors	78	7.6
Six risk Factors	57	5.5
Seven risk Factors	43	4.2
Eight risk Factors	35	3.4
Nine risk Factors	22	2.1
Ten risk Factors	46	4.5
**Identification of Stroke symptoms**		
Zero	593	57.6
One symptom	36	3.5
Two symptoms	13	1.3
Three symptoms	61	5.9
Four symptoms	74	7.2
Five symptoms	60	5.8
Six symptoms	51	5
Seven symptoms	141	13.7
**Consequences Identified**		
Zero	450	43.7
One Problem	241	23.4
Two Problems	167	16.2
Three problems	88	8.6
Four Problems	83	8.1

**Table 3:** Logistic regression to determine the association of stroke risk factors, early symptoms, and consequences with participants’ characteristics.

Variables	OR (95% CI)	*p*-Value
**Risk factor(s) identified (≥1)**		
Gender (male versus female *)	1.34 (0.81–2.23)	0.26
Residence area (rural versus urban *)	1.60 (0.85–3.01)	0.15
Region (Central Region versus Western Region *)	**2.42 (1.56–3.75)**	**0.001**
Region (Eastern Region versus Western Region *)	0.69 (0.47–1.03)	0.07
Region (Northern Region versus Western Region *)	1.57 (0.89–2.77)	0.12
Region (Southern Region versus Western Region *)	1.45 (0.78–2.69)	0.25
Educational level (school versus university *)	0.92 (0.61–1.37)	0.68
Employment status (employed versus unemployed *)	1.06 (0.75–1.49)	0.74
History of smoking (Current smoker versus Ex-smoker *)	**1.95 (1.38–2.75)**	**0.001**
Type of Smoking (Cigarette versus E-cigarette *)	1.40 (0.98–2.00)	0.07
Type of Smoking (Shisha versus E-cigarette *)	1.16 (0.79–1.72)	0.45
**Identified Stroke Symptoms (≥1)**		
Gender (male versus female *)	**2.88 (1.86–4.47)**	**0.001**
Residence area (rural versus urban *)	1.18 (0.63–2.20)	0.61
Region (Central Region versus Western Region *)	0.71 (0.46–1.09)	0.11
Region (Eastern Region versus Western Region *)	1.14 (0.81–1.61)	0.45
Region (Northern Region versus Western Region *)	0.65 (0.38–1.11)	0.11
Region (Southern Region versus Western Region *)	0.78 (0.44–1.39)	0.41
Educational level (school versus university *)	**0.53 (0.37–0.76)**	**0.001**
Employment status (employed versus unemployed *)	1.09 (0.80–1.48)	0.59
History of smoking (Current smoker versus Ex-smoker *)	**2.68 (2.00–3.59)**	**0.001**
Type of Smoking (Cigarette versus E-cigarette *)	0.99 (0.71–1.38)	0.96
Type of Smoking (Shisha versus E-cigarette *)	0.93 (0.65–1.32)	0.68
**Identified consequences (≥1)**		
Gender (male versus female *)	0.76 (0.49–1.17)	0.21
Residence area (rural versus urban *)	1.12 (0.60–2.09)	0.72
Region (Central Region versus Western Region *)	**3.16 (2.05–4.88)**	**0.001**
Region (Eastern Region versus Western Region *)	**1.52 (1.08–2.14)**	**0.02**
Region (Northern Region versus Western Region *)	0.80 (0.45–1.41)	0.43
Region (Southern Region versus Western Region *)	1.81 (1.01–3.25)	0.05
Educational level (school versus university *)	**0.64 (0.43–0.93)**	**0.02**
Employment status (employed versus unemployed *)	**1.40 (1.03–1.91)**	**0.03**
History of smoking (Current smoker versus Ex-smoker *)	**2.36 (1.73–3.21)**	**0.001**
Type of Smoking (Cigarette versus E-cigarette *)	**2.01 (1.45–2.79)**	**0.001**
Type of Smoking (Shisha versus E-cigarette *)	**0.63 (0.44–0.91)**	**0.01**

* Reference variable.

## Data Availability

The original contributions presented in this study are included in the article. Further inquiries can be directed to the corresponding author.
